# A proof of concept study on digital interventions for reducing socio-evaluative stress and anxiety in youth

**DOI:** 10.1038/s41598-025-96602-6

**Published:** 2025-04-11

**Authors:** Rüya Akdağ, Mariska E. Kret, Evin Aktar, Milica Nikolić

**Affiliations:** 1 Department of Cognitive Psychology, Institute of Psychology, Leiden University, Wassenaarseweg 52, 2333 AK Leiden, The Netherlands; 2 Leiden Institute for Brain and Cognition (LIBC), Leiden University, Leiden, The Netherlands; 3 Department of Clinical Psychology, Institute of Psychology, Leiden University, Leiden, The Netherlands; 4 Research Institute of Child Development and Education, University of Amsterdam, Amsterdam, The Netherlands

**Keywords:** Social evaluative threat, Subclinical social anxiety, Heart rate variability, Negative metacognitive thoughts, Digital interventions, Psychology, Human behaviour

## Abstract

Youth often struggle with heightened sensitivity to social judgement, increasing their vulnerability to fear in social situations. This study investigates brief digital interventions aimed at regulating cognitive and affective disturbances related to social anxiety, specifically focusing on fear responses in a social-evaluative threat context. One-hundred-twenty healthy youth were randomly assigned to one of four conditions: attention training, detached mindfulness, slow breathing, or an active control condition. To induce social-evaluative threat, participants delivered a public speech after a 12-min video intervention. We assessed subjective anxiety levels, metacognition, heart rate variability (HRV), and subjective and objective performance ratings throughout the experiment. Results indicated that the slow breathing intervention significantly increased HRV immediately after the intervention, during the public speaking task, and during the recovery and reduced state anxiety immediately after the intervention. In contrast, attention training and detached mindfulness did not yield significant effects, although detached mindfulness did increase HRV immediately post-intervention. These preliminary findings suggest that brief digital interventions, especially slow breathing, may effectively alleviate fear responses in youth during social-evaluative contexts, highlighting their potential as accessible support tools.

**Supplementary Information:**

The online version contains supplementary material available at 10.1038/s41598-025-96602-6.

## Introduction

In today’s world of expanding social networks and interconnectedness, social anxiety is a common issue that affects people across cultures, ages, and backgrounds. We are all familiar with the apprehensions we experience before voicing our opinions in a group setting or the hesitations accompanying unfamiliar social encounters. For approximately 10% of the Western population, these common fears can escalate into uncontrollable anxiety affecting their daily lives, resulting in social anxiety disorder (SAD), a severe mental health problem that has doubled since the pandemic^1^. However, for a much larger portion of the population, shyness and nervousness in social situations pose significant challenges that often remain untreated^2^. This problem is especially persistent during adolescence when social anxiety peaks due to a strong self-focus and concern about others’ evaluations^3^. Consequently, many adolescents fear being judged negatively when they are the center of attention and frequently report being shy and nervous around others^4^. These fears remain prevalent and persistent^5–7^. For example, social anxiety levels reach a peak in subclinical youth in the period between 18 and 24 years of age^8^. Based on the definition by the United Nations, we refer to this age period as “youth”^9^.

Individuals with heightened social anxiety experience cognitive and affective disturbances before, during, and after stressful social situations. Specifically, in the cognitive domain, maladaptive metacognition or too much “thinking about thinking” has recently been in focus in relation to both subclinical and clinical levels of social anxiety^10–12^. During social interactions, metacognition shifts the attention to the self and increases the focus on worrying^10^ which is related to feelings of lowered confidence in social skills^13^. In the affective domain, disturbances in emotion regulation reflected in reduced heart rate variability (HRV)^14^ have also been shown in relation to subclinical and clinical levels of social anxiety^15,16^.

The Neurovisceral Integration Model provides a theoretical framework linking metacognition, HRV, and emotional regulation^17^. This model posits that higher-order cognitive processes, such as metacognition, engage prefrontal cortex activity, which in turn modulates autonomic nervous system regulation. When maladaptive metacognitive processes dominate, prefrontal inhibitory control over the autonomic system weakens, leading to reduced HRV and heightened emotional dysregulation^18,19^. While this interplay is well-documented in the context of clinical anxiety, its mechanisms in subclinical youth remain less understood. Additionally, we know little about how to alleviate these disturbances in social-evaluative situations in youth.

Despite the availability of treatments for SAD, a significant number of socially anxious youth tend to avoid seeking help, primarily due to their fear of social exposure and anticipation of negative evaluations^20^. Consequently, social anxiety often remains undertreated emphasizing the imperative need to develop easily accessible interventions. One way to achieve this is through digital interventions, as they do not require direct involvement from therapists, thereby reducing social exposure. Many mental health apps exist, but they often lack evidence-based techniques that tackle core impairments, making them potentially harmful^21^. Studies examining evidence-based online interventions have demonstrated that they hold promise but often involve therapist or parent participation^22^. Few studies have examined the effectiveness of digital self-help interventions, which are especially valuable for youth with subclinical levels of social anxiety who struggle with their symptoms but avoid treatment due to their fear of others’ evaluations.

Therefore, in this proof-of-concept study, we aimed to investigate the potential of converting evidence-based treatments related to metacognition and HRV into a digital format for youth with varying levels of social anxiety. By addressing both metacognition and HRV in our study, we can get a more comprehensive view about the underlying symptoms of social anxiety and how to treat them. The objective of this proof-of-concept study is to explore the efficacy of these interventions in a stand-alone brief digital format on subsequent socio-evaluative stress responses during a socially threatening situation of giving a public speech. Ultimately, the overarching vision for this intervention is to be delivered in a mobile application over time, considering its accessibility and appeal to youth^23^ and investigating longer-term effects on socio-evaluative stress responses.

We developed brief video interventions based on in-person techniques known to regulate metacognition and heart rate variability (HRV). To tackle negative metacognitions, attention training and detached mindfulness, both included in the metacognitive therapy designed by Wells^24^, have been previously used. Attention training focuses on relearning attention flexibility to redirect the focus from negative metacognitive thoughts, whereas detached mindfulness enhances the sense of inner-awareness of negative thoughts using the acceptance of thoughts rather than the active analyses of them^24^. Both attention training and detached mindfulness, within the context of metacognitive therapy, are known to improve anxiety and depression that are sustained over a longer period of time (e.g., 3–24 months)^25^. However, both interventions have recently been tested as stand-alone and more short-term treatments. Specifically, attention training has been shown to decrease negative metacognitive thoughts when given as a stand-alone treatment in adults^26,27^. Similarly, detached mindfulness was effective as a stand-alone treatment by lowering anxiety levels in adults^28^. These findings suggest that both interventions may affect metacognition not only in the long term but also in the short term. Importantly, apart from the individual use of these interventions, there are several reasons why they are well suited for a digital format. Specifically, (1) the exercises are performed independently without the interference of a therapist, (2) the elaborate instruction protocols can be easily standardized and translated in a video and audio instruction, and lastly (3) the structured nature of the inventions involve clear, step-by-step procedures that can be directly replicated in a digital environment without losing its effectiveness.

To target HRV, we employed the slow breathing technique, which is known to increase HRV, thereby enhancing autonomic regulation, a critical process in modulating fear responses and emotional regulation^29^. Slow breathing encourages a specific breathing pattern with longer exhalation periods compared to inhalation. This form of breathing is suggested to activate parasympathetic activity, enhancing relaxation^30,31^. The effectiveness of increasing HRV has been repeatedly shown, and previous presentations of the technique in a digital format proved to be well-suited as a digital intervention^32^. Similarly to detached mindfulness, recent research indicates that sustained practice of slow breathing, spanning over an extended duration (e.g., 12 weeks), results in significant enhancement of cardiovascular parameters and reduction in perceived stress levels^33^. This suggests that the benefits of slow breathing also extend beyond immediate effect, indicating its potential to confer longer-term improvement.

Thus, as a first step, in our proof-of-concept study we investigated the immediate effects of our digital interventions on the cognitive and affective mechanisms they are intended to target. Eventually this could contribute to the understanding of effective treatments for anxiety disorders. We investigated this during a social-evaluative threat in youth between 18 and 24 years of age. Participants were randomly assigned to one of the three interventions or the control group after we induced social-evaluative stress by informing them that they would need to give a public speech. After the intervention, all participants prepared and delivered a public speech to an audience (which the participants were led to believe was live but was actually pre-recorded), during which we assessed their metacognition, HRV, and social anxiety before, during, and after the speech. We hypothesized that (1) attention training and detached mindfulness would reduce negative metacognitions before, during, and after public speaking compared to the control conditions, (2) slow breathing would increase HRV before, during, and after public speaking compared to the control group, (3) all three interventions would lower state anxiety before, during, and after public speaking compared to the control group, and (4) all three interventions would lead to better subjective and objective performance evaluations in the public speaking task than those in the control condition.

## Results

### Preliminary analyses

#### Randomization

To check whether our randomization led to equal groups, we compared all demographic characteristics and traits between participants in different conditions. No significant differences between conditions were found in age, gender, education distribution, or trait metacognition (all *p* values > .104). However, there was a significant difference in trait levels of social anxiety between conditions *F*(3, 120) = 3.513, *p* = .018. On average, participants scored 40.41 on trait social anxiety, which according to the Social Phobia and Anxiety Inventory (SPAI), reflects elevated social anxiety levels within our non-clinical sample^34^. Notably, participants in the detached mindfulness condition reported significantly higher on social anxiety (*M* = 47.292, *SD* = 15.991) than those in the attention training condition (*M* = 34.228, *SD* = 14.125), while no significant differences were found between the other conditions (all *p* values > .054). Importantly, the scores on state metacognition, state anxiety, and state HRV—our main outcomes—did not significantly differ between the conditions in the baseline and pre-intervention phases (all *p* values > .070). See Supplementary Table 1 for a full overview of all the descriptive statistics.

#### Stress induction check

Participants experienced higher levels of anxiety during the pre-intervention stress induction phase, when we announced the public speaking task, compared to the baseline phase (*Z* = − 7.655, *p* < .001), confirming the effective induction of social anxiety prior to the intervention.

### Main analyses

#### State metacognition

**Fig. 1:**
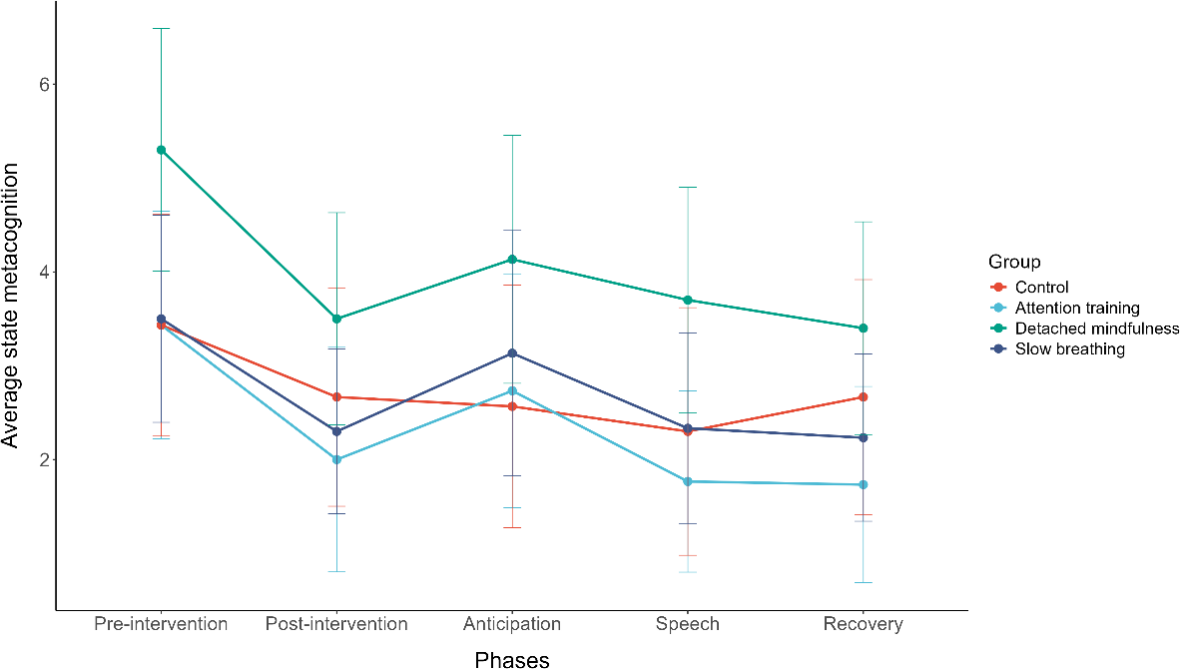
Metacognition state from pre-intervention to recovery for different conditions. The higher the score on metacognition, the more negative metacognitive beliefs a participant has.

In the first model, we tested the effects of the interventions on state metacognition from pre-intervention to post-intervention, anticipation, public speech, and recovery, accounting for the baseline scores on state metacognition. Accounting for the baseline state metacognition, we only found a significant main effect of phase *F*(4, 480) = 30.364, *p* ≤ .001. Condition, *F*(3, 120) = 0.810, *p* = .490, and the interaction between condition and phase, *F*(12, 480) = 1.377, *p* = .173, were not significantly predicting state metacognition. See Table 1 and Fig. 1 for an overview of those findings.

**Table 1:** Results of the linear model predicting state metacognition by condition, phase, and their interaction while correcting for baseline metacognition (N = 120).

	Estimate	Std. error	df	t value	*p* value
Condition category					
Attention training	− 0.000	0.400	365.512	− 0.000	1.000
Detached mindfulness	0.281	0.404	356.854	0.695	.488
Slow breathing	0.010	0.400	365.501	0.025	.980
Phase category					
Post-intervention	− 0.767	0.310	480.000	− 2.474	.014*
Anticipation	− 0.867	0.310	480.000	− 2.797	.005**
Speech	− 1.133	0.310	480.000	− 3.658	< .001***
Recovery	− 0.767	0.310	480.000	− 2.474	.014*
Baseline scores	0.850	0.031	120.000	27.444	< .001***
Interactions					
Attention training*post-intervention	− 0.667	0.438	480.000	− 1.521	.129
Detached mindfulness*post-intervention	− 1.033	0.438	480.000	− 2.358	.019*
Slow breathing*post-intervention	− 0.433	0.438	480.000	− 0.989	.323
Attention training*anticipation	0.167	0.438	480.000	0.380	.704
Detached mindfulness*anticipation	− 0.300	0.438	480.000	− 0.685	.494
Slow breathing*anticipation	0.500	0.438	480.000	1.141	.254
Attention training*speech	− 0.533	0.438	480.000	− 1.217	.224
Detached mindfulness*speech	− 0.467	0.438	480.000	− 1.065	.287
Slow breathing*speech	− 0.033	0.438	480.000	− 0.076	.939
Attention training*recovery	− 0.933	0.438	480.000	− 2.130	.034*
Detached mindfulness*recovery	− 1.133	0.438	480.000	− 2.586	.010*
Slow breathing*recovery	− 0.500	0.438	480.000	− 1.141	.254

The control condition and pre-intervention phase are the reference categories.

*p < .05, **p < .01, ***p < .001.

#### State HRV

**Fig. 2:**
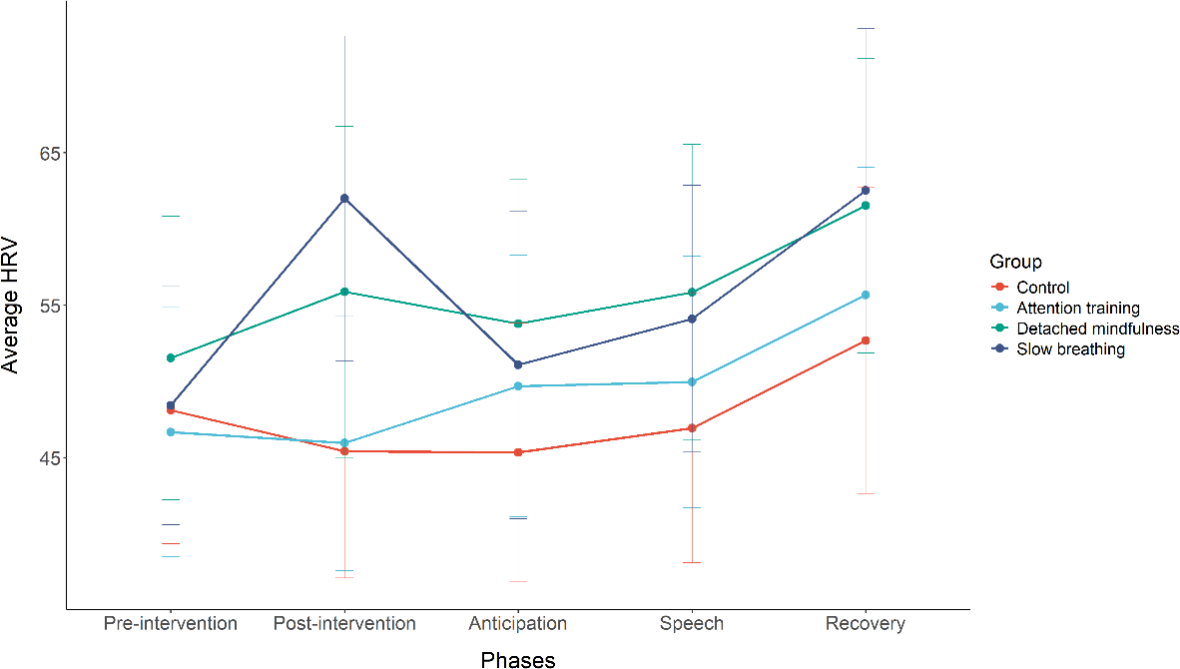
HRV from pre-intervention to recovery for different conditions. The higher the score on HRV, the better the physiological regulation.

In the second model, we tested the effects of the interventions on HRV from pre-intervention to post-intervention, anticipation, public speech, and recovery, accounting for the baseline scores on state HRV and respiration rate. Accounting for baseline HRV, we found a significant main effect of phase *F*(4, 468) = 16.677, *p* ≤ .001. The interaction between condition and phase was also significant, *F*(12, 468) = 2.803, *p* ≤ .001. The main effect of condition *F*(3, 117) = 2.482, *p* = .064 and respiration rate *F*(1, 553) = 0.001, *p* = .978 were not significant. As expected, participants in the slow breathing intervention group showed a significantly higher increase in HRV from pre-intervention to post-intervention, speech, and recovery phases compared to the control condition (see Table 2 and Fig. 2 HRV scores in the detached mindfulness condition also significantly increased from pre-intervention to post-intervention compared to the control condition (see Table 2). No other differences between the conditions were observed.

**Table 2:** Results of the linear model predicting HRV by condition category, phase category, and their interaction while correcting for baseline HRV (N = 117).

	Estimate	Std. error	df	t value	*p* value
Condition category					
Attention training	0.812	3.480	286	0.233	.722
Detached mindfulness	− 0.381	3.443	284	− 0.111	.914
Slow breathing	− 0.377	3.413	285	− 0.111	.916
Phase category					
Post-intervention	− 2.708	2.414	471	− 1.122	.285
Anticipation	− 2.562	2.391	469	− 1.072	.266
Speech	− 1.190	2.387	469	− 0.499	.635
Recovery	4.565	2.384	469	1.915	.065
Baseline score	− 0.001	0.030	550	− 0.030	< .001***
Respiration rate	0.713	0.032	118	22.059	.979
Interactions					
Attention training*post-intervention	2.416	3.427	468	0.705	.580
Detached mindfulness*post-intervention	7.031	3.392	468	2.073	.047*
Slow breathing*post-intervention	15.964	3.367	468	4.742	< .000***
Attention training*anticipation	6.011	3.424	468	1.755	.104
Detached mindfulness*anticipation	4.801	3.392	468	1.415	.155
Slow breathing*anticipation	4.563	3.363	468	1.357	.121
Attention training*speech	4.920	3.423	468	1.437	.208
Detached mindfulness*speech	5.492	3.387	468	1.621	.119
Slow breathing*speech	6.861	3.362	468	2.041	.050*
Attention training*recovery	4.870	3.420	468	1.424	.212
Detached mindfulness*recovery	5.423	3.387	468	1.601	.124
Slow breathing*recovery	9.519	3.359	468	2.834	.007**

The control condition and anticipation phase are the reference categories.

*p < .05, **p < .01, ***p < .001.

#### State anxiety

**Fig. 3:**
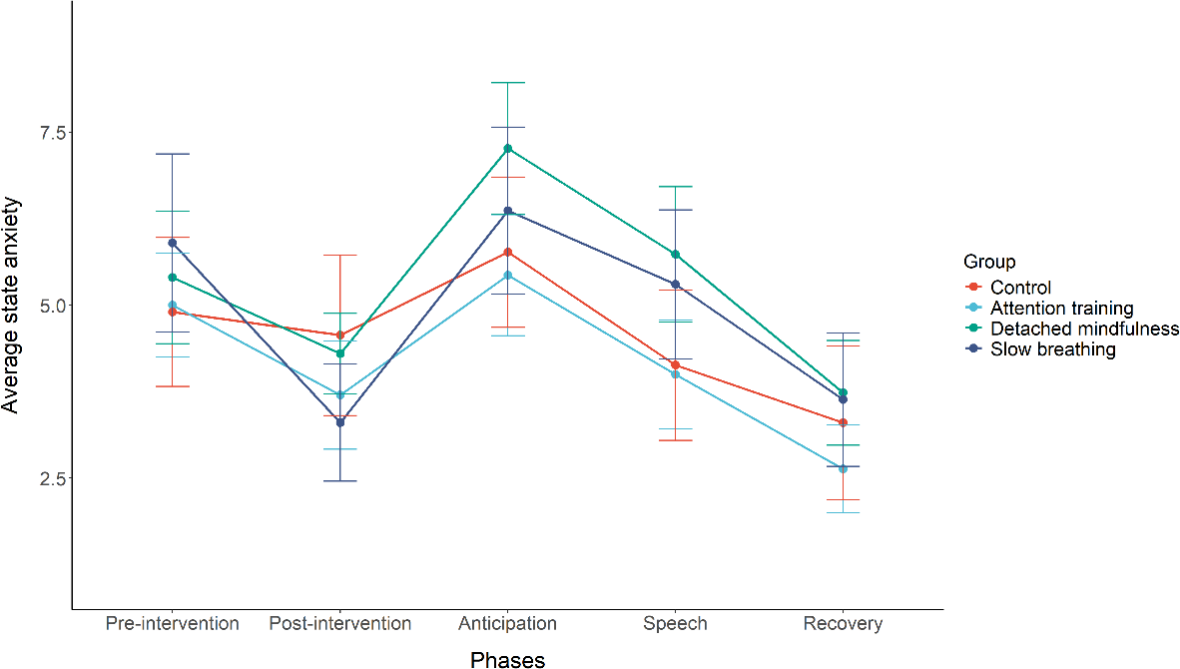
State anxiety from pre-intervention to recovery for different conditions. The higher the score on anxiety, the more anxious a participant is.

In the third multilevel model, we tested the effects of the interventions on state anxiety from pre-intervention to post-intervention, anticipation, public speech, and recovery. Accounting for the baseline scores on state anxiety, we found a significant main effect of phase *F*(4, 480) = 54.405, *p* < .001, and the interaction between condition and phase *F*(12, 480) = 2.635, *p* = .002. The main effect of condition was not significant *F*(3, 240) = 1.189, *p* = .317. Interestingly, only for the slow breathing intervention group state anxiety significantly decreased from pre- to post-intervention compared to the control condition (see Table 3 and Fig. 3).

**Table 3:** Results of the linear model predicting state anxiety by condition category, phase category, and their interaction while correcting for baseline anxiety (N = 120).

	Estimate	Std. error	df	t value	*p* value
Condition category					
Attention training	− 0.158	0.591	321	− 0.268	.789
Detached mindfulness	− 0.073	0.593	319	− 0.123	.902
Slow breathing	− 1.078	0.591	321	− 1.825	.069
Phase category					
Post-intervention	0.333	0.432	480	0.772	.440
Anticipation	− 0.867	0.432	480	− 2.007	.045*
Speech	0.767	0.432	480	1.776	.076
Recovery	1.600	0.432	480	3.706	< .001***
Baseline scores	0.582	0.068	120	8.533	< .001***
Interactions					
Attention training*post-intervention	0.967	0.611	480	1.583	.114
Detached mindfulness*post-intervention	0.767	0.611	480	1.256	.210
Slow breathing*post-intervention	2.267	0.611	480	3.712	< .001***
Attention training*anticipation	0.433	0.611	480	0.710	.478
Detached mindfulness*anticipation	− 1.000	0.611	480	− 1.638	.102
Slow breathing*anticipation	0.400	0.611	480	0.655	.513
Attention training*speech	0.233	0.611	480	0.382	.703
Detached mindfulness*speech	− 1.100	0.611	480	− 1.802	.072
Slow breathing*speech	− 0.167	0.611	480	− 0.273	.785
Attention training*recovery	0.767	0.611	480	1.256	.210
Detached mindfulness*recovery	0.067	0.611	480	0.109	.913
Slow breathing*recovery	0.667	0.611	480	1.092	.275

The control condition and the anticipation phase are the reference categories.

*p < .05, **p < .01, ***p < .001.

#### Performance

In the last model, we tested the effects of the interventions on subjective performance during anticipation and public speech. We found no significant effects of condition *F*(3, 120) = 1.355, *p* = .260, phase *F*(1, 120) = 2.777, *p* = .098, and the interaction between phase and condition *F*(3, 120) = 0.449, *p* = .718. Thus, participants in all intervention conditions did not differ in their subjective performance ratings from the control group (see Supplementary Table 2). Similarly, in the model with objective performance as an outcome, there were no significant differences between the conditions *F*(3, 117) = 2.255, *p* = .086.

## Discussion

The present study examined whether evidence-based short digital interventions could reduce metacognition, increase HRV, and reduce social anxiety symptoms in youth when faced with a social-evaluative threat. The results revealed that immediately after the intervention, our slow breathing intervention increased HRV and reduced state anxiety. This effect was also found during and after the public speech for HRV. However, we did not find evidence that detached mindfulness and attention training affected negative metacognitive beliefs or state anxiety.

In line with our expectations, slow breathing was more effective in increasing HRV compared to the control condition. This finding is in line with previous research showing that a slow breathing intervention is able to increase HRV^14^. Our study demonstrates that even one short 12-min intervention can enhance HRV, and interestingly, this effect, although small in size, also remains during the socially stressful public speech and immediately after it. These findings highlight the suitability of the slow breathing intervention for social stress reduction during social-evaluative threat in youth. Surprisingly, the detached mindfulness group also showed increased HRV immediately after the intervention. Although detached mindfulness is thought to primarily target negative thoughts^28^, our findings raise the possibility that detached mindfulness influences emotion regulation reflected in increased HRV. This finding corresponds to more recent research that shows that mindfulness-based interventions can increase HRV^35,36^. A possible mechanism that could be at play is that detached mindfulness might enhance self-compassion^37^. While individuals practicing detached mindfulness may experience stress and worry, they are encouraged to acknowledge and accept their thoughts as transient phenomena. Additionally, they are encouraged to look at their negative thoughts in a non-judgmental manner, thereby enhancing the sense of self-compassion^24^. Self-compassion has been consistently shown to be associated with higher levels of HRV^38,39^. Despite the fact that we found an association between detached mindfulness and HRV, more research is required to delve into the nuanced interplay between detached mindfulness and its effects on parasympathetic activation, paving the way for a more comprehensive understanding of mindfulness interventions.

We did not find significant effects of detached mindfulness and attention training interventions on metacognition. These interventions did not lead to a decrease in negative metacognitive beliefs compared to the control group. This finding was against our hypothesis. There could be a few explanations as to why the effects were not strong enough in our sample. First of all, compared to the slow breathing intervention, detached mindfulness has more complicated instructions. It might, therefore, be more challenging to understand the concept and the exercise after performing only one intervention session. This explanation is supported by the finding that participants in our sample rated the detached mindfulness condition as significantly more difficult to understand compared to the attention training and slow breathing conditions (see Supplementary Results 2). Interestingly, attention training was not rated as more difficult than slow breathing. However, the participants in our sample disliked this intervention the most compared to the slow breathing and detached mindfulness intervention (which was liked the most).

A second explanation could be that our sample, which was mostly composed of non-socially anxious individuals, scored low on average in negative metacognitive beliefs. These interventions are specifically designed to target altered metacognitive mechanisms. However, when these altered mechanisms are not highly activated, the study might lack the power to capture any small improvements. Lastly, detached mindfulness allows participants to practice dealing with negative thoughts that individuals might experience when giving a presentation. However, in this proof-of-concept study, we used generalized and standardized thoughts for everyone to practice with. Consequently, some participants might have found the thoughts irrelevant to them and, therefore, did not fully engage with the exercise. For future studies, we suggest providing more detailed instructions on how to perform the detached mindfulness intervention and incorporating individual-specific thoughts. Such specific instructions could facilitate better understanding of the intervention, making it more guided. Additionally, adding an adherence measure to the exercise could significantly enhance understanding of the working mechanisms of the interventions.

All three intervention groups showed a similar pattern in state social anxiety as the control group, except during the post-intervention phase. The slow breathing intervention significantly reduced state anxiety compared to the control group immediately following the intervention. This finding aligns with previous studies that have demonstrated reductions in anxiety and arousal after single sessions of slow breathing^40^. In contrast, metacognitive interventions had not been previously introduced as a single brief intervention, and our study is among the first to present them in this format. It is plausible that metacognitive interventions yield effects on state anxiety after multiple sessions, possibly due to their more complex nature. 

Furthermore, our study showed that none of the interventions effectively reduced state anxiety before, during, and after the socially stressful speech. Thus, while the effects of interventions such as slow breathing may be apparent immediately after the intervention and in the absence of a social-evaluative threat, influencing anxiety levels during social-evaluative threat situations appears to be more challenging. It is possible that a single short intervention may not be sufficient for alleviating anxiety in an actual stressful situation, and repeated exercise of the same digital intervention could prove more beneficial in managing long-term and stressful situations.

We did not observe any effects of interventions on the objective and subjective performance measures. The similar performance across groups implies that public speaking performance may be relatively inflexible and resistant to change following just a single intervention session. Additionally, the chosen intervention do not directly target performance and this might be the reason as to why one session might not entail the desired results. Regardless, in our exploratory analyses (Supplemental Material), we observed an effect of trait social anxiety on the subjective but not on the objective performance rating in a way that higher social anxiety trait levels were related to lower ratings for self-reported performance. This finding aligns with previous research demonstrating that socially anxious individuals receive similar ratings from objective observers as non-socially anxious individuals but tend to underestimate their own performance compared to non-socially anxious individuals^41^.

The results of this study should be interpreted in light of some limitations. First, the control condition was created to match the multimedia characteristics of the video interventions. Nonetheless, it is advisable for future research to identify appropriate control conditions for slow breathing and metacognitive interventions separately. Second, the study predominantly involved Western female students, limiting the generalizability of the findings to males, younger adolescents, and youth from diverse cultural backgrounds. Notably, social anxiety symptoms can vary across cultures, with a higher prevalence in Eastern cultures^42^. Additionally, in the Netherlands, there is an alarming dropout rate from therapy of youth with immigration backgrounds, specifically at 75%^43^. Reasons for dropout are frequently associated with a sense of not being understood or an inability to establish a connection with therapists, who often possess a Western background. Therefore, it is highly relevant to investigate the effects of our interventions in diverse cultural settings, as digital interventions possess the unique capability to transcend cultural barriers and tailor their approach to the specific requirements of various underrepresented populations^44^. Third, in the absence of any previous data to inform more precise power analyses, we conducted an a priori power analysis based on a MANOVA repeated measures design to ensure sufficient power to detect moderate interaction effects between condition and phase. We acknowledge that these power calculations do not correspond precisely to the performed analyses. Ideally, a simulation based approach would be used to calculate a needed sample size for our multi-level models. Now that we have collected first empirical data, future similar studies can use these findings to conduct more precise sample size estimations to ensure sufficient power to further validate and extend these findings. Last, although the primary objective was to assess whether the digital interventions effectively address the cognitive and affective symptoms they were designed for, future well-powered studies should explore whether the changes in these cognitive and affective symptoms act as mechanisms of change in social anxiety through mediation analyses.

Nonetheless, this study boasts several strengths. First, it examines the impact of brief digital intervention on cognitive and affective disturbances within a real-life, socially challenging event. Second, a comprehensive array of measures was employed to evaluate stress-related symptoms, including self-reports, physiological indices, and objective behavior measures. Third, the present study represents an initial exploration into the potential of implementing evidence-based interventions in a digital format to address social stress issues among both healthy youth and those exhibiting subclinical levels of social anxiety. Consequently, future research should aim to investigate the effectiveness of evidence-based digital interventions, both in the short term and long term, in mitigating social anxiety-related disturbances in real-life situations among subclinical samples of youth. Additionally, understanding whether the timing of the intervention can reduce stress response and better prepare individuals remains an important direction for future studies. More specifically, whether adjusting the timing of the intervention—delivering it before the anticipation or just before the speech phase—further enhances its effectiveness.

In summary, the results of this study demonstrate that it is possible to reduce psychobiological social stress responses in a real-life, socially stressful situation involving healthy youth after just one session of a brief, evidence-based digital intervention. Therefore, in this preliminary step, digital interventions show promise for addressing common social fears. This highlights the importance of further developing and examining these interventions to potentially help undiagnosed youth who experience shyness and nervousness in social situations to effectively cope with socially challenging events.

## Methods

### Ethical approval

The study was approved by the Psychology Research Ethics Committee at Leiden University (2022-11-14-M.E. Kret-V1-4339). The research procedure complies with the ethical standards of the national and institutional committees and is in accordance with Helsinki Declaration. Additionally, the hypotheses, preprocessing of the data and statistical analyses of the study were pre-registered (https://osf.io/hx63u/?view_only=84b331dcf3944a0ea7298f1f5181f511).

### Participants

This study involved 120 healthy Dutch undergraduate students from Leiden University aged 18–24 years (*M* = 19.65, 111 female). Participants were randomly assigned to one of the four conditions: 30 to attention training, 30 to detached mindfulness, 30 to slow breathing, and 30 to active control.

As this was a proof-of-concept study, and we lacked any prior data that could inform more precise power calculations for our multilevel models, we opted to conduct an apriori power analysis using G*Power MANOVA design with a within-between-subject interaction (in which we were specifically interested as per our pre-registration) to estimate a needed sample size. Noteworthy, the power analysis with MANOVA provides a rather conservative estimation. As pre-registered, we ran multilevel models which are recognized for their heightened statistical power when compared to MANOVA^45^.

The analysis included one between-subject factor (condition) with 4 levels, and one within-subjects factor (phase) with 6 levels. Using power of 0.80, alpha level α = 0.05 and a medium effect size of 0.25 (drawing on effect sizes reported in prior intervention studies targeting comparable mechanisms^46,47^), the analysis showed that we needed 105 subjects in total. With our sample we, therefore, had enough power to detect the expected interaction effect. Additionally, to determine at what phases the intervention effects on the outcome would appear, we conducted power analyses for our planned comparisons. Specifically, we tested each intervention group against the control group at each post-intervention phase, using pre-intervention as the reference point (i.e., simple effects). For this, we again ran G*Power power analyses but for an ANOVA repeated measures with within- and between-subject interaction to estimate the needed sample size for planned comparisons. For each simple effect, we planned to compare two groups (one intervention and one control) at two time points (e.g., post-intervention versus pre-intervention). Therefore, we ran this analysis for two groups and two measurement points. Considering we were interested in 12 comparisons, we used the Bonferroni correction for multiple testing leading to an adjusted alpha level of 0.004 (0.05/12). Using a medium effect size (η^2^ = 0.25) and power of 0.80, the required sample size was 30 participants per group (120 participants in total). Therefore, we had enough power to detect planned comparisons with our sample.

### Procedure

Participants were recruited through SONA, an online screening tool for student participation at Leiden University. During the pre-screening process, participants reviewed the list of enrollment criteria including any past or current psychological or neurological disorders, normal or corrected vision, and proficient Dutch comprehension (C1), before signing the consent form. Participants who completed the informed consent were invited to a single lab session at Leiden University. Prior to the session, they were instructed to avoid heavy physical activity and alcohol consumption one day before the experiment and heavy meals and caffeinated drinks two hours before the session to control for transient variables influencing HRV values^40^.

Upon arrival at the lab, participants received a comprehensive briefing and were requested to read and sign the informed consent form. Following this, they completed a questionnaire about their trait social anxiety and trait metacognitive levels. Participants were also asked to note how many hours they slept the night before and whether this number deviated from their average sleep quality, as poor sleep can adversely affect HRV^48^. Additionally, we collected data on participants’ familiarity with mindfulness practices to account for possible performance variations linked to daily practices. The check for both variables between groups can be found in Supplementary Results 1.

#### Public speaking task

**Fig. 4:**
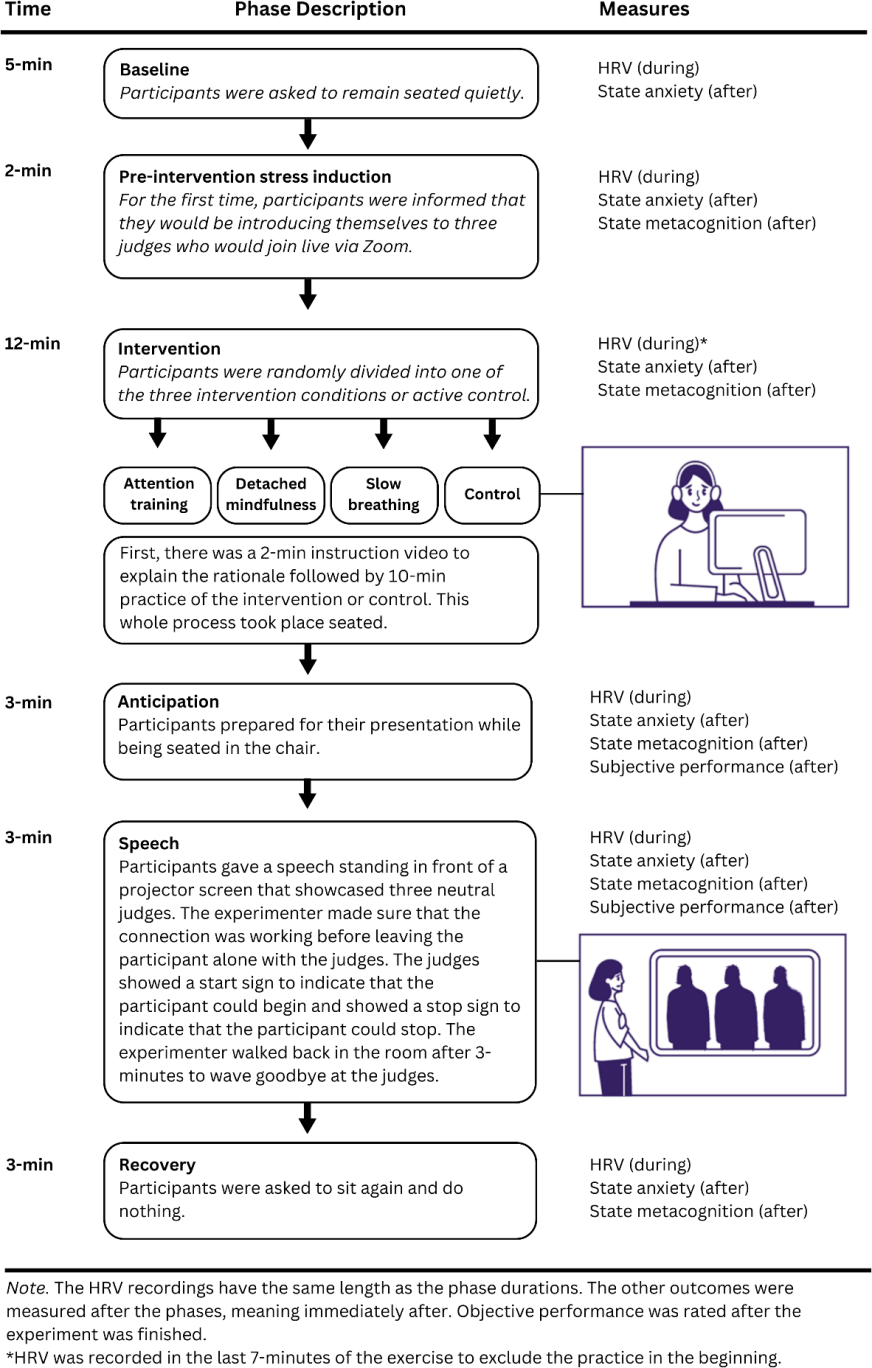
Overview of the public speaking task procedure.

For the public speaking task, we utilized an adapted version of the Leiden Public Speaking Task, recognized for inducing nervousness and physical anxiety responses in adolescents^49^. The study procedure encompassed six phases: (1) baseline, (2) pre-intervention stress induction, (3) post-intervention, (4) anticipation, (5) speech, and (6) recovery; a concise overview is presented in Fig. 4. Participants started with a 5-min baseline HRV measurement, connected to the wireless BIOPAC R-SPEC BioNomadix module for heart rate and respiration monitoring. Subsequently, participants were briefed by the experimenter, introducing the self-introduction in front of three judges (pre-intervention stress induction), during which HRV, state anxiety, and state metacognition were measured, lasting a total of 2 min. We introduced the public speaking task before the intervention to elicit the activation of negative metacognitive thoughts and decreased HRV.

Subsequently, participants were randomly assigned to one of the three interventions or the control condition. They began by viewing a 2-min instructional video explaining the rationale behind the intervention. Following this, participants rated the perceived helpfulness of the intervention in reducing anxiety on a scale from − 3 “not helpful at all” to 3 “completely helpful”*,* with 0 indicating neutrality. This allowed us to assess differences in perceived effectiveness before the actual intervention. The intervention itself was then practiced for 10 min. For each intervention, we inquired about participants’ understanding and likeability of the intervention and feasibility of practicing the exercises daily. The data for these variables can be found in Supplementary Results 2. Immediately after the intervention, we measured self-reported state anxiety and metacognition (post-intervention). Then, the anticipation phase started, during which participants were given 3 min to prepare how they would introduce themselves. At the end of the anticipation, we once again measured self-reported state anxiety and state metacognition.

Following the anticipation, the 3-min speech phase started. Participants stood in front of a projector screen, where they were informed about the live connection with the judges. The experimenter started the pre-recorded video featuring three judges entering a room and taking their seats. A prompt was given to the experimenter to ask the judges whether they could hear and see us. The judges responded, and the participant began their presentation when signaled to start by the judges and concluded when the judges signaled to stop. Throughout the presentation, the judges remained neutral and silent. After the 3-min presentation, the judges showed a stop sign, and the experimenter entered the room to inform them that they could now score the presentation and waved goodbye. These prompts between the experimenter and the pre-recorded judges were included to create a realistic illusion. Approximately 95% of participants believed the judges were present, while the remaining 5% (six participants) were skeptical about the judges. We asked each of the six participants why they were skeptical and five of them explained that because of experienced deception in prior psychology studies they were more skeptical. The one participants explained that they just had a feeling it was not reel. Running the analyses without these participants yielded similar results, hence why we kept them in the main analyses. Following the speech phase, participants reported their state anxiety and state metacognition. Subsequently, the participants were instructed to sit and relax for three minutes in the recovery phase.

The main outcome measures, state anxiety and HRV, were measured during all six phases, and state metacognition was measured from pre-intervention to recovery because it pertained to the speech introduced during the pre-intervention stress induction. Participants remained seated in front of a computer for all phases except the speech, for which they stood in front of the life-size judges’ screen. The speech phase began after participants had stood up, positioned themselves in front of the screen, and initiated their presentations. Following the recovery phase, the electrodes were removed, and participants received a comprehensive debriefing about the experiment and the deception. The entire experiment lasted 80 min.

### Variables

#### Manipulated variable: intervention condition

We randomly assigned participants to engage in one of three interventions: attention training, detached mindfulness, and slow breathing, or the control video. Each video was standardized to include both audio and video inputs. We used this multimedia format of showing animations and audio instructions because youth have reportedly pointed out to find this kind of content more engaging and easier to follow than written text or audio alone^50^. The interventions are available at https://osf.io/ej9by/?view_only=29436f64b58d4f7782e8fcd6906307a8.

##### Attention training technique

Attention training is an auditory listening technique involving selective attention, attention switching, and divided attention^51^. Individuals are initially instructed to listen to specific sounds while disregarding others (i.e., selective attention) for the first 5 min. They are then instructed to rapidly switch their focus of attention between sounds (i.e., attention switching) for the next 5 min and finally are instructed to focus on multiple sounds at once (i.e., divided attention) for 2 min.

##### Detached mindfulness

The main idea of detached mindfulness is that participants learn that they do not have to interact with negative thoughts and learn to see them as noise in the mind. We created this video using four of the ten detached mindfulness techniques as described by Adrian Wells^24^. We divided the techniques into two parts: (1) the explanation part, including the suppression-counter suppression, busy road metaphor, and the observing self, and (2) the practice part, including the free-association task.

##### Slow breathing

Slow breathing was presented as a 12-min exercise video. Participants watched an animation that showed them how long to (1) breathe in, (2) hold their breath, and (3) breathe out. We adapted a commonly used slow breathing video^32^. Based on previous studies, they were instructed to breathe from the abdomen at a rate of six breaths per minute and with a higher exhale-to-inhale ratio^52,53^.

##### Control condition

A video where participants listened to the Wikipedia page about giraffes while viewing a screen with a blurred image of giraffes behind a fixation dot was found to be a non-arousing control compared to emotional stories^54^.

#### Measured variables

##### Self-reported trait social anxiety

The shortened and translated version of the Social Phobia and Anxiety Inventory (SPAI, translated in Dutch)^55,56^, the SPAI-18^34^, was used to measure the experienced level of social anxiety symptoms. Participants indicated for 18 items on a scale from 1 to 7 how often they experience the described thoughts and feelings in social situations, 1 being “never” and 7 being “always”. An example question is “I feel tense when I have to give a speech in front of an audience”. The entire SPAI-18 has an excellent internal consistency of 0.93 for healthy populations. In our sample, the interreliability was also excellent (*α* = 0.975). The sum of all items was computed per participant.

##### Self-reported trait metacognition

To measure negative metacognition, we used the translated version of the metacognition questionnaire—adolescents (MCQ-A translated in Dutch)^57,58^. Participants have to indicate for 30 items on a scale from 1 to 4 how much they agree with statements about their thoughts. 1 being “do not agree” and 4 being “completely agree”. For example, “Worrying is bad for me”. The internal consistency for the MCQA is good at 0.88. In our sample, the interreliability was also good (*α* = 0.868). The sum of all items was computed per participant.

#### Main outcome variables

##### State social anxiety

We used the shortened and translated version of the State Trait Anxiety Inventory-S (STAI-S)^59^ to measure how anxious participants felt during the six phases. Participants indicated on six items how they feel right now on a scale from 1 to 4, with 1 being “completely not” and 4 being “very much”. For example, “I feel calm”. For each phase, the sum of the six items was computed per participant. The average was then taken of all the participants per condition to obtain one composite score per condition.

##### State metacognition

We created an adapted version based on the MCQ-A. We used three items from the negative beliefs subscale and two from the cognitive confidence subscale and adjusted them to relate to the speech performance. These two subscales were chosen because they are known to be altered in social anxiety^11^. For example. “My worrying about the speech makes me uncomfortable.” We formatted the questionnaire similarly to the state anxiety questionnaire. The internal consistency analyses revealed an eigenvalue greater than 1 (3.384) with a variance of 67.68%. The state metacognition questionnaire measured one factor where all items had large positive loadings (all above 0.720). We computed the sum of all items per participant and later averaged all the participant scores per condition.

##### Heart rate variability and respiration

HRV was retrieved as RMSSD, a common metric for HRV^60^, using the PhysioDataToolbox Version 0.6.3^61^. RMSSD was calculated for the six epochs corresponding to our six phases using the interbeat interval (IBI), which was derived from heart rate (HR) data. While HR was not analyzed separately, it was used to calculate RMSSD. Although we initially intended to include high-frequency heart rate variability (HRV-HF) in our analysis, we decided against it due to the potential confounding effects of the slow breathing intervention. Measuring HRV-HF during slow breathing can artificially suppress variability because the breathing rate often falls below the frequency range typically associated with HRV-HF (0.15–0.4 Hz). This synchronization between heart rate and slow breathing can dampen the natural fluctuations in heart rate, leading to lower HRV-HF readings and potentially misleading interpretations regarding autonomic function. Therefore, we opted to focus on RMSSD as a more appropriate metric for assessing heart rate variability in this context. For all six epochs, RMSSD was included as an absolute score rather than a difference score. Additionally, baseline RMSSD was included as a reference in the statistical models mentioned below to account for individual differences at baseline.

The respiration rate was calculated by dividing the number of breaths by the epoch duration. The RMSSD and respiration rates were averaged for all participants per condition. Participants were asked to remain seated for all but the speech phase. To ensure no overlapping effects from the movement transition, we only measured the HRV in the speech phase after they moved and were standing still, having time to acclimate to their standing position. 

##### Subjective performance

We adapted the Expected and Evaluated Performance Questionnaire^62,63^ to measure how participants thought they would and believed they performed a speech in front of the judges. Instead of relating the questions to adolescents and the teacher used in their video, we related the questions to the judges in our video. The questions are answered on a 1–5 scale, with 1 being, for example, “worse than most others” or “very bad” and 5 being “better than most others” or “very good”. For example, “how well do you think you will give the presentation compared to your peers?”. The sum scores were calculated for each participant in the anticipation and speech phase and later averaged per condition.

##### Objective performance

We used the adapted and translated version of the performance questionnaire^41^ to assess objective speech performance. This questionnaire is also translated into Dutch^62,64^. Observers rated participants’ performance on 11 items scaled from 1 to 4, with 1 being “very much” and 4 being “not at all”. For example, “how loud and clear was the voice of the speaker. To ensure reliability, two raters independently assessed the first 10% of cases, achieving a strong interrater reliability score of 0.82. The videos were then randomly divided over the raters including an equal amount of each of the conditions. After this randomization the videos were blinded. The scores of the two raters were averaged for each condition.

Subsequently, one rater evaluated the remaining 90% and computed individual sum scores. These individual sum scores were then averaged within each condition.

### Statistical analyses

All data were analyzed using RStudio 2022.07.0. We checked the assumptions for our main outcome variables: (1) state anxiety, (2) state metacognition, (3) state HRV, (4) subjective performance ratings, and (5) objective performance ratings. Data normality was checked through histograms and the Shapiro‒Wilk test. State metacognition and HRV data exhibited non-normal distributions, leading us to employ the Kendall-Theil test for regression analysis. State anxiety deviated slightly from normality in the baseline and pre-intervention phases and thus was analyzed using the Wilcoxon signed rank test for baseline data. Both subjective and objective performance data followed a normal distribution, and all variables exhibited linear relationships, as confirmed by scatterplots.

#### Preliminary analyses

First, we assessed the success of randomization by comparing participant demographics and traits across conditions. We used a MANOVA for trait anxiety and trait metacognition. Within the randomization, we also checked if there were differences between state anxiety, state metacognition, and HRV using a Mann–Whitney U test. The Mann–Whitney U test was used because our data followed a non-normal distribution, hence, nonparametric testing is more fitting. In line with the pre-registration, we verified the induction of subjective state anxiety during the pre-intervention stress induction phase by comparing baseline and pre-intervention anxiety. However, because the distribution of our data was non-normal, we opted for the nonparametric equivalent of the paired sample t-test, namely, the Wilcoxon signed-rank test. We omitted the manipulation check because the values are now added to the main analyses. See below for an explanation of this change in the analysis plan. Finally, we assessed possible ceiling effects and identified which participants benefited most from the interventions by performing regressions of the difference scores of our dependent variables. The data for the regression analyses can be found in Supplementary Results 3.

#### Main analyses

We included three separate mixed-effects linear models to investigate the impact of interventions on the dependent variables: state metacognition, HRV, and state anxiety, considering different social stress phases from pre-intervention stress induction. All main analyses followed the pre-registration with one exception: pre-intervention scores, which were initially pre-registered as a covariate, were used as a reference category for all other (post-intervention) phases. This choice aimed to acknowledge that the pre-intervention scores were not independent variables but part of the nested structure of variables that were repeatedly observer over time. Using them as a reference category allowed us to assess whether there were changes following the intervention from the pre-intervention scores. Our change was also supported by two independent expert who did not have insights into our data and results.

In the first multilevel model, state metacognition served as the dependent variable. We included three independent variables: (1) condition, a between-subject variable encompassing the conditions attention training, detached mindfulness, slow breathing, and control, (2) phase, a within-subject variable covering the pre-intervention, post-intervention, anticipation, speech, and recovery phases, and (3) the interaction between condition and phase. The models encompassed fixed effects for condition, phase, their interactions, and random intercepts for each participant related to the dependent variable. Inclusion of random slopes for the predictors condition and phase was initially planned, as outlined in our pre-registration. However, model convergence issues arose during estimation, indicating that the dataset did not provide sufficient information to support the more complex random-effects structure. Given the risk of unreliable parameter estimates, we opted to exclude random slopes and retain only random intercepts, ensuring a more stable and interpretable model.

The second and third models mirrored the first but with distinct dependent variables and corresponding covariates. For the second model, HRV served as the dependent variable, and respiration was added as an additional control variable. In the third model, state anxiety was the dependent variable. For the second HRV model, we additionally included the baseline HRV scores to account for baseline differences. Similarly, for the anxiety model, we included the baseline state anxiety scores. All models incorporated clustering to nest measurements of the dependent variables across the five phases within individuals.

For our main analyses, when the interaction was significant, we compared the intervention conditions to the control, with the control condition serving as the reference category. For phase, we compared all the phases to the pre-intervention phase. In all our analyses, we used a Bonferroni correction for our post-hoc pairwise comparisons between the factor levels and their interactions in case of significant model terms.

To predict subjective performance ratings, we conducted a multilevel model, focusing solely on the anticipation and speech phase and including condition as independent variables. The model also included fixed effects for condition and phase and their interactions and random intercepts for each participant related to the dependent variable.

For the objective speech performance ratings, we computed an ANOVA, as the performance was only rated once for each participant after the experiment. The ANOVA included objective performance rating as the dependent variable and condition as the between-subject variable.

#### Exploratory analyses

We examined potential differences between participants with higher and lower scores on trait social anxiety within our healthy sample. We included the interaction effect between social anxiety trait levels and condition in all our statistical models. Additionally, we investigated potential differences between participants with higher and lower scores on trait metacognition to predict state metacognition. To do so, we added the interaction effect between trait metacognition and condition only to the first model. These analyses are reported in Supplementary Results 4.

#### Missing data

We conducted data quality checks to address issues related to faulty data entry, processing, and poor sampling. No missing data were found for state anxiety, state metacognition, and subjective performance scores. However, for objective performance, two participants lacked video recordings due to a file-saving error, and one participant’s file became corrupted. Consequently, we excluded these three participants from the objective performance analysis, resulting in a sample size of *N* = 117, detached mindfulness consisting of *N* = 28 and slow breathing of *N* = 29. For HRV analysis, two participants experienced significant movement artifacts that distorted their HRV values, and one participant’s heart rate data were incomplete due to BIOPAC issues. Consequently, these three participants were excluded from the HRV analysis, leaving us with a total of* N* = 117 participants, attention training consisting of *N* = 28 participants and detached mindfulness consisting of *N* = 29.

#### Assumption checks and outliers

We checked assumptions for the linear multilevel models by plotting residuals against fitted values and identifying outliers, defined as data points located three standard deviations from the mean. We identified seven outliers in the HRV values, so we ran analyses where we removed the extreme outliers by winsorizing them. However, the results remained highly similar. Hence, we decided to keep the model with all data points because they were all within no extraordinary boundaries.

## Electronic supplementary material

Below is the link to the electronic supplementary material.

Supplementary Material 1

## Data Availability

The pre-processed data and code to analyze the data are accessible through the Open Science Framework following this link https://osf.io/ej9by/.
