# Supplementary material for: A proof of concept study on digital interventions for reducing socio-evaluative stress and anxiety in youth
Source: Sci Rep. 2025 Apr 11;15:12417. doi: 10.1038/s41598-025-96602-6 (PMC11992151; doi:10.1038/s41598-025-96602-6)
Supplement: Supplementary file 1 — Supplementary Material 1 [file 41598_2025_96602_MOESM1_ESM.docx]

**Supplementary materials**

Supplementary Tables

**Supplementary Table 1. Descriptives Statistics Table of All Outcome Variables**

|  |  | **State anxiety** | | **State metacognition** | | **HRV** | | **Subjective**  **Performance** | | **Objective**  **Performance** | |
| --- | --- | --- | --- | --- | --- | --- | --- | --- | --- | --- | --- |
| **Overall model** | **Phases** | Mean | SD | Mean | SD | Mean | SD | Mean | SD | Mean | SD |
|  | Baseline | 11.208 | 2.351 | - | - | 55.884 | 29.858 | - | - | - | - |
|  | Pre-intervention | 9.700 | 2.886 | 3.917 | 3.407 | 48.706 | 23.342 | - | - | - | - |
|  | Post-intervention | 11.033 | 2.446 | 2.617 | 3.085 | 52.394 | 27.208 | - | - | - | - |
|  | Anticipation | 8.792 | 2.947 | 3.142 | 3.617 | 49.953 | 25.279 | 16.083 | 3.108 | - | - |
|  | Speech | 10.208 | 2.831 | 2.525 | 3.210 | 51.715 | 24.532 | 16.417 | 3.118 | 32.923 | 3.721 |
|  | Recovery | 11.675 | 2.481 | 2.508 | 3.065 | 58.123 | 26,910 | - | - | - | - |
| **Control** |  | | | | | | | | | | |
|  | Baseline | 11.33 | 2.057 | - | - | 55.213 | 32.297 | - | - | - | - |
|  | Pre-intervention | 10.10 | 3.010 | 3.43 | 3.308 | 48.117 | 24.513 | - | - | - | - |
|  | Post-intervention | 10.43 | 3.2455 | 2.62 | 3.299 | 45.422 | 23.233 | - | - | - | - |
|  | Anticipation | 9.233 | 2.445 | 2.567 | 3.483 | 45.347 | 23.763 | 16.600 | 3.979 | - | - |
|  | Speech | 10.867 | 2.197 | 2.300 | 2.700 | 46.934 | 24.706 | 16.600 | 4.132 | 34.033 | 2.895 |
|  | Recovery | 11.700 | 1.771 | 2.667 | 2.924 | 52.689 | 28.052 | - | - | - | - |
| **Attention training** |  |  |  |  |  |  |  |  |  |  |  |
|  | Baseline | 11.43 | 2.079 | - | - | 51,438 | 26,664 | - | - | - | - |
|  | Pre-intervention | 10.00 | 2.101 | 3.43 | 3.390 | 46.676 | 22.099 | - | - | - | - |
|  | Post-intervention | 11.30 | 2.184 | 2.00 | 3.343 | 45.957 | 22.568 | - | - | - | - |
|  | Anticipation | 9.567 | 3.036 | 2.733 | 3.607 | 49.694 | 23.171 | 16.400 | 3.001 | - | - |
|  | Speech | 11.000 | 3.037 | 1.767 | 3.687 | 49.975 | 22.291 | 16.967 | 2.189 | 32.033 | 4.255 |
|  | Recovery | 12.367 | 3.098 | 1.733 | 3.497 | 55.683 | 22.615 | - | - | - | - |
| **Detached mindfulness** |  |  |  |  |  |  |  |  |  |  |  |
|  | Baseline | 10.60 | 2.621 | - | - | 60.552 | 28.979 | - | - | - | - |
|  | Pre-intervention | 9.60 | 2.596 | 5.30 | 3.612 | 51.548 | 25.578 | - | - | - | - |
|  | Post-intervention | 10.70 | 1.622 | 3.50 | 3.160 | 55.878 | 29.875 | - | - | - | - |
|  | Anticipation | 7.733 | 2.651 | 4.133 | 3.693 | 53.790 | 26.101 | 15.100 | 2.746 | - | - |
|  | Speech | 9.267 | 2.741 | 3.700 | 3.354 | 55.856 | 26.668 | 15.633 | 3.243 | 33.536 | 3.372 |
|  | Recovery | 11.267 | 2.116 | 3.400 | 3.169 | 61.541 | 26.580 | - | - | - | - |
| **Slow breathing** |  |  |  |  |  |  |  |  |  |  |  |
|  | Baseline | 11.47 | 2.696 | - | - | 56.190 | 31.762 | - | - | - | - |
|  | Pre-intervention | 9.10 | 3.595 | 3.50 | 3.02 | 48.442 | 21.902 | - | - | - | - |
|  | Post-intervention | 11.70 | 2.366 | 2.30 | 2.452 | 62.006 | 29.788 | - | - | - | - |
|  | Anticipation | 8.633 | 3.378 | 3.133 | 3.655 | 51.093 | 28.208 | 16.233 | 2.417 | - | - |
|  | Speech | 9.700 | 3.007 | 2.333 | 2.832 | 54.115 | 24.454 | 16.467 | 2.556 | 32.103 | 3.967 |
|  | Recovery | 11.367 | 2.697 | 2.233 | 2.487 | 62.531 | 29.722 | - | - | - | - |

*Note.* The table shows the main outcome variables' means and standard deviations per phase. First for the overall model and later per group. For state anxiety, state metacognition, and subjective performance, the data was summarized over 120 participants in total. For HRV and objective performance, the data were summarized over 117 participants.

**Supplementary Table 2. Results of the Linear Model Predicting Subjective Performance by Group Category, Phase Category, and Their Interaction (*N* = 120)**

|  | Estimate | Std. Error | df | t-value | p-value |
| --- | --- | --- | --- | --- | --- |
| **Group category** |  |  |  |  |  |
| Attention training | -0.200 | 0.788 | 154.700 | -0.254 | .800 |
| Detached mindfulness | -1.500 | 0.788 | 154.700 | -1.903 | .059 |
| Slow breathing | -0.367 | 0.788 | 154.700 | -0.465 | .642 |
| **Phase category** |  |  |  |  |  |
| Speech | -0.000 | 0.400 | 120.000 | 0.000 | 1.000 |
| **Interactions** |  |  |  |  |  |
| Attention training*speech | 0.567 | 0.566 | 120.000 | 1.002 | .319 |
| Detached mindfulness*speech | 0.533 | 0.566 | 120.000 | 0.943 | .348 |
| Slow breathing*speech | 0.233 | 0.566 | 120.000 | 0.412 | .681 |

*Note.* The control group and anticipation phase are the reference categories.
* p = .05, ** p < .01, *** p < .001

Supplementary Results

Supplementary Results 1. Sleep quality and familiarity with the interventions

We conducted frequency analyses for both variables and compared them between groups. For sleep quality, participants could indicate how many hours they slept the night before and if that was normal or more than usual (scores as 2) or less than usual (scored as 1). For familiarity, we asked whether they practiced mindfulness/yoga/meditation never, weekly, monthly, or never (scores as 1 to 4, respectively). The frequency distribution for both variables can be found in Supplementary Table 3. We performed two separate chi-square tests to examine whether the distributions differed significantly between groups. The chi-square for sleep quality revealed no significant difference *X*^2^ (3, *N* = 120) = 6.046, *p* = .109. The chi-square for familiarity also revealed no significant difference *X*^2^ (9, *N* = 120) = 7.954, *p* = .539. Thus, there were no differences between the groups related to sleep quality and familiarity with the exercises.

**Supplementary Table 3. Frequencies for Sleep Quality and Familiarity Distributions of the Four Groups (*N* = 120)**

|  |  | Sleep Quality | | Familiarity | |
| --- | --- | --- | --- | --- | --- |
|  |  | Frequency | Percent | Frequency | Percent |
| CT | 1 | 3 | 10 | 23 | 76.7 |
|  | 2 | 27 | 90 | 3 | 10.0 |
|  | 3 | - | - | 3 | 13.3 |
|  | 4 | - | - | 1 | 3.3 |
| ATT | 1 | 9 | 30 | 24 | 80 |
|  | 2 | 21 | 70 | 4 | 13.3 |
|  | 3 | - | - | 2 | 6.7 |
| DM | 1 | 11 | 36.7 | 22 | 73.3 |
|  | 2 | 19 | 63.3 | 6 | 20 |
|  | 3 | - | - | 2 | 6.7 |
| SB | 1 | 8 | 26.7 | 18 | 60 |
|  | 2 | 22 | 73.3 | 7 | 23.3 |
|  | 3 | - | - | 5 | 16.7 |

Supplementary Results 2. Differences in credibility, feasibility, likeability, and understandability

After viewing the 2-minute instructional video during the intervention phase, participants rated the perceived helpfulness of the intervention in reducing anxiety on a scale from -3 "not helpful at all" to 3 "completely helpful"*,* with 0 indicating neutrality. This allowed us to assess differences in perceived effectiveness before the actual intervention. Furthermore, we also measured afterward for each intervention the participants' understanding of the instructions, the likeability of the intervention, and the feasibility of practicing the exercises daily, similar to credibility. Thus, -3 indicates "completely not likable, feasible, or understandable," and 3 "completely likable, feasible, and understandable," with again 0 indicating neutrality.

We ran four separate ANOVAs to measure differences between attention training, detached mindfulness, and slow breathing. In Supplementary Table 4, you can find an overview of the descriptives and F-test results. The results revealed only a significant difference between the three interventions for understandability. Post-hoc comparisons specified that there was a difference between detached mindfulness and attention training (diff = -0.900, *p* < .001) and detached mindfulness and slow breathing (diff = 0.767, *p* < .001). More specifically, detached mindfulness was the most difficult to understand compared to attention training and slow breathing.

|  | Attention training | | Detached mindfulness | | Slow breathing | | F(2, 87) | η^2^ |
| --- | --- | --- | --- | --- | --- | --- | --- | --- |
| Measures | M | SD | M | SD | M | SD |  |  |
| Credibility | 1.067 | 0.583 | 1.300 | 0.702 | 1.47 | 0.629 | 2.957 | 0.063 |
| Likeability | 0.167 | 1.289 | 0.900 | 1.242 | 0.800 | 1.375 | 2.795 | 0.060 |
| Feasability | -0.067 | 1.574 | 0.567 | 1.654 | 0.000 | 1.762 | 1.31 | 0.029 |
| Understandability | 2.567 | 0.568 | 1.667 | 0.959 | 2.433 | 0.774 | 11.53*** | 0.210 |
| * p = .05, ** p < .01, *** p < .001 | | | | | | | | |

**Supplementary Table 4. Descriptive Statistics and the Results of the Four ANOVAs for Each Dependent Variable Separately with Condition Category as Between-subject (*N* =90)**

Supplementary Results 3. Regression models to predict differences in intervention effects

We assessed possible ceiling effects and identified which participants benefited most from the interventions by performing regressions of the difference scores of our dependent variables by calculating change scores for state anxiety, HRV, and state metacognition from pre-intervention to post-intervention. We computed a linear regression for state anxiety and the robust non-parametric Kendall-Theil regression for metacognition and HRV. In all models, we added the change scores as predictors and state anxiety, metacognition, and HRV as dependent variables.

The regression models revealed that the change in symptoms from pre-intervention to post-intervention was predicted by the pre-intervention score for that symptom in such a way that participants who were scoring higher at the pre-intervention on state anxiety (*B =* 0.401, *p* < .001) and state metacognition (*Estimate =* .375, *p* < .001) also had a higher reduction in symptoms after the intervention. For HRV no such effect was observed (*Estimate =* -0.026, *p* = .452).

Supplementary Results 4. The added interaction with the trait scores

**State metacognition**

The added interaction effect between social anxiety trait levels and condition was not significant when predicting state metacognition *F*(4, 120) = 2.258, *p =* .067. The added interaction effect between metacognition trait level and condition was also not significant when predicting state metacognition *F*(4, 120) = 0.580, *p =* .678.

**State HRV**

The added interaction effect between social anxiety trait levels and condition was not significant when predicting state HRV *F*(4, 117 = 0.665, *p =* .618).

**State anxiety**

Notably, the added interaction effect between social anxiety trait and the condition was significant *F*(4, 120) = 10.448, *p* < .001. The individual estimates revealed a significant interaction between the control condition and social anxiety trait (*t* = 0.071, *p* < .001), attention training and social anxiety trait (*t* = 0.043, *p* = .031), and between the slow breathing and social anxiety trait (*t* = 0.079, *p* < .001). The findings indicated that participants with higher social anxiety trait levels in the control, attention training, and slow breathing conditions experienced higher state anxiety than those with lower social anxiety trait levels.

**Performance**

The added interaction effect between social anxiety trait and condition was significant for predicting subjective performance *F*(4, 120) = 15.970, *p* < .001. The individual estimates revealed a significant interaction for all conditions (all *p*-values below .02). Specifically, more socially anxious participants judged themselves poorer on their speech performance compared to less socially anxious participants. Adding the social anxiety trait scores as a covariate for the objective performance did not yield a significant effect (*F*(1, 120) = 0.973, *p* = .326). Thus, there were no significant differences in objective performance ratings between individuals who are more or less socially anxious.
